# The Use of Oseltamivir as Adjunctive Therapy for the Treatment of Hand-Food-and-Mouth Disease: A Meta-Analysis of Randomized Clinical Trials

**DOI:** 10.3389/fphar.2021.653691

**Published:** 2021-06-25

**Authors:** Yijing Zhao, Yangyang Sun, Raphael N. Alolga, Gaoxiang Ma, Fan Wang

**Affiliations:** ^1^ The Clinical Metabolomics Center, School of Traditional Chinese Pharmacy, China Pharmaceutical University, Nanjing, China; ^2^ State Key Laboratory of Natural Medicines, School of Traditional Chinese Pharmacy, China Pharmaceutical University, Nanjing, China; ^3^ School of International Pharmaceutical Business, China Pharmaceutical University, Nanjing, China

**Keywords:** hand-foot-and-mouth disease, oseltamivir, adjunctive therapy, efficacy, randomized clinical trials

## Abstract

**Background:** Hand-foot-and-mouth disease (HFMD) is a common childhood illness caused by enteroviruses. Oseltamivir (OS), a neuraminidase inhibitor, has been frequently used as an adjunctive therapy for the treatment of HFMD. Solid evidence, however, is lacking regarding the efficacy of such adjunctive therapy. This work is to conduct a meta-analysis of randomized clinical trials (RCTs) to assess the efficacy of oseltamivir for HFMD in children.

**Methods:** Eligible studies from inception to October 10, 2020 were identified by searching six databases (PubMed, Embase, CENTRAL, CNKI, Wanfang, and VIP database). Quality of evidence was assessed using the Cochrane Collaboration tool.

**Results:** Of a total of 91 entries, 11 RCTs involving 977 HFMD children were included in the final analysis. The results showed that the therapy combined with oseltamivir was more effective, with higher effective rate (RR, 0.84; 95% CI, 0.80 to 0.87; *p* < 0.01), shorter fever clearance time (days) (SMD, −0.74; 95% CI, −1.12 to −0.35; *p* < 0.01), shorter rash regression time (days) (MD, −0.89; 95% CI, −1.05 to −0.72; *p* < 0.01) and shorter clinical cure time (SMD, −1.08; 95% CI, −1.55 to −0.61; *p* < 0.01). No significant difference was observed in the risk of adverse reactions between the groups with and without oseltamivir.

**Conclusion:** The use of oseltamivir as adjunctive therapy shows effectiveness and no increased risk of adverse reactions for the treatment of HFMD in children.

## Introduction

Hand-foot-and-mouth disease (HFMD) is a common infectious disorder caused most by enterovirus A71 (EV71) and coxsackievirus A16 (CV-A16), and both can cause epidemic disease (Xing et al., 2014; Li et al., 2018). The enterovirus can spread through coughs and sneezes of infected people. Children younger than 5 years are susceptible to infection of HFMD. Most of patients have mild, self-limiting illness typically including fever, rashes on hands and feet, and mouth ulcers. The illness usually runs its course in seven to ten days (Cox and Levent, 2018). A small proportion of infected children may rapidly develop severe and even fatal neurological and systemic complications over days (Xing et al., 2014). Antiviral drugs such as ribavirin (RBV), vidarabine monophosphate (ara-AMP), and acyclovir are usually recommended to treat HFMD.

Oseltamivir (OS), a neuraminidase inhibitor, has been widely used against influenza virus infection by inhibiting virus replication (Treanor et al., 2000; Dobson et al., 2015; Malosh et al., 2018). At present, OS is increasingly used as adjunctive therapy in combination with other medications for the treatment of HFMD in China. Many studies have shown that the combination therapy is effective against the HFMD. It can shorten the course of the disease and alleviate symptoms without obvious adverse effects. This study aims to assess the efficacy of OS in adjuvant treatment of HFMD based on published randomized controlled trials (RCTs).

## Material and Methods

### Data Sources and Search Strategy

Studies were identified through searches in six electronic databases [PubMed, EMBASE, Cochrane database, the China National Knowledge Infrastructure (CNKI), Wanfang database, and VIP database] up to Octorber 2020. The search terms were “oseltamivir” and “hand, foot, and mouth disease” (both text words and Medical Subject Headings terms). The CNKI, Wanfang, and VIP database provided literature in Chinese. The Chinese search terms were “奥司他韦” and “手足口病”.

### Study Selection and Data Extraction

Studies included met the following criteria: 1) they were randomized clinical trials; 2) they were quantitative researches; 3) patients in such studies were diagnosed according to the Guidelines for the Diagnosis and Treatment of HFMD; 4) treatment with a combination of oseltamivir and conventional therapy. Two authors assessed the study eligibility and extracted the data independently. Any disagreement was resolved through consensus. A predefined form was used to extract data from each study. Primary outcomes were the total effective rate, fever clearance time, rash regression time, clinical cure time, and incidence of adverse events.

### Risk of Bias Assessment

The quality of the included studies was assessed by two authors using the Cochrane Collaboration’s tool: random sequence generation (selection bias), allocation concealment (selection bias), blinding of participants and personnel (performance bias), blinding of outcome assessment (detection bias), incomplete outcome data (attrition bias), selective reporting (reporting bias), and other bias.

### Statistical Analysis

Data analyses were performed using Review Manager (version 5.4.1; Cochrane Collaboration) software. Risk ratios (RR) were combined for dichotomous data, and mean differences (MD) or standardized mean differences (SMD) for continuous data. The heterogeneity among studies was assessed with the Cochran’ Q and *I* ^*2*^ statistic. If heterogeneity (Q-test’s *p* < 0·05 or *I* ^*2*^ > 50%) was observed, the random-effect model was applied, otherwise, fixed-effect model was used. Subgroup analysis stratified by different drug treatments was carried out. Funnel plots were planned to assess the publication bias. The sensitivity analysis was conducted after sequentially excluding each study from the meta-analysis. In addition, publication bias was addressed by a Begg’s rank correlation, an Egger’s regression, and a trim-and-fill method.

## Results

**FIGURE 1:**
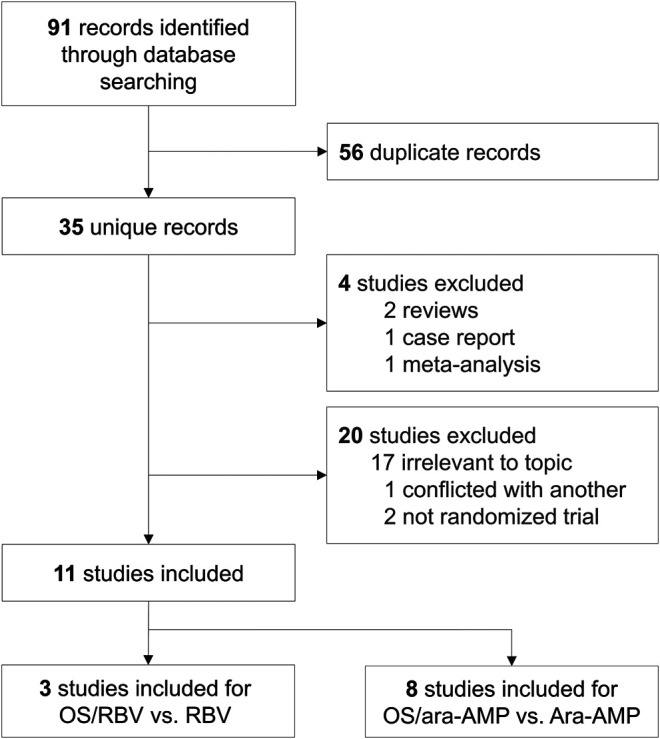
Flow chart of study selection.

The initial search identified 91 studies, of which 36 were included after exclusion of duplicates. After comprehensive evaluation, 25 studies were excluded for not meeting the inclusion criteria. 11 RCTs were included in the final meta-analysis (Cai et al., 2013; Xu, 2016; Zeng, 2016; Zhou and Hu, 2016; Li, 2018; Lu, 2018; Shen, 2018; Si et al., 2018; Tong, 2018; Geng et al., 2020; Zhu, 2020), of which three studies were for comparison of observation group receiving OS combined with RBV (OS/RBV group) and control group receiving RBV therapy alone (RBV group) (Cai et al., 2013; Xu, 2016; Zeng, 2016). Eight studies reported data on the comparison of OS/ara-AMP group and ara-AMP group (Zhou and Hu, 2016; Li, 2018; Lu, 2018; Shen, 2018; Si et al., 2018; Tong, 2018; Geng et al., 2020; Zhu, 2020) (Figure 1).

### Characteristics of the Eligible Studies

The study characteristics are summarized in Table 1. The total number of patients involved was 977. The number of patients in each trail ranged from 23 to 70. The comparison of OS/RBV (192 patients) vs. RBV (192 patients) was investigated in three studies, and OS/ara-AMP (297 patients) vs. Ara-AMP (296 patients) in eight studies. The studies were published between 2013 and 2020. The onset time of the enrolled patients in each trail ranged from 0.5 to 9 days.

**TABLE 1:** Characteristics of the studies included in the meta-analysis.

Study id	Study period	Age, y	Onset	Course, d	Treatment		Sample size			Outcome
			time, d		Experimental	Control	Experimental	Control	Total	Indicators
Cai et al. (2013)	2012/4–2012/7	1–5	3	5	OS/RBV	RBV	60	60	120	a b c
Xu (2016)	2014/5–2015/5	1–5	1–2	5	OS/RBV	RBV	62	62	124	a
Zeng (2016)	2015/10–2016/3	1–5	3–5	5	OS/RBV	RBV	70	70	140	a b c
Geng et al. (2020)	2017/6–2018/4	1–5	2–10	10	OS/ara-AMP	Ara-AMP	50	50	100	a b c d e
Li (2018)	2017/1–2018/3	1–5	0.5–7	10	OS/ara-AMP	Ara-AMP	40	40	80	a
Lu (2018)	2015/7–2017/4	0.5–5	4.5–5.5	10	OS/ara-AMP	Ara-AMP	45	45	90	a b c d
Shen (2018)	2017/3–2018/3	2–6	1–8	5	OS/ara-AMP	Ara-AMP	33	33	66	a
Si et al. (2018)	2014/2–2016/12	2–5	0.8–7.5	10	OS/ara-AMP	Ara-AMP	30	30	60	a b c d e
Tong (2018)	2016/3–2018/3	1–5	1.5–7	5	OS/ara-AMP	Ara-AMP	23	23	46	a b c d e
Zhou and Hu (2016)	2013/8–2015/6	0.8–6	−	10	OS/ara-AMP	Ara-AMP	32	32	64	a b c d e
Zhu (2020)	2017/6–2019/6	1–5	1–9	10	OS/ara-AMP	Ara-AMP	44	43	87	a

OS = oseltamivir; RBV = ribavirin; ara-AMP = vidarabine monophosphate.

a Total effective rate.

b Fever clearance time.

c Rash regression time.

d Clinical cure time.

e Adverse reaction rate.

### Risk of Bias Assessment

**FIGURE 2:**
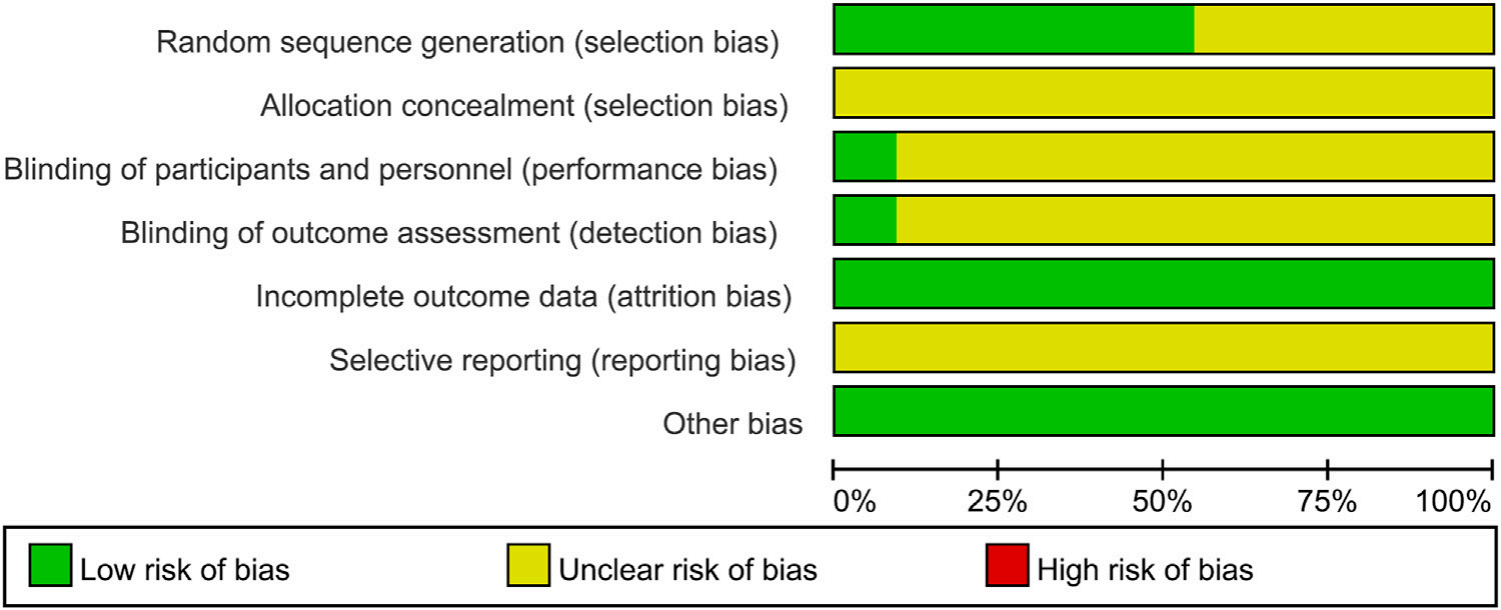
Risk of bias assessment for studies included using the Cochrane Collaboration’s tool.

The majority of studies did not describe the methodology in sufficient detail. Hence, the risk of bias of most of the studies was generally unclear (Figure 2). There were six studies describing the random distribution method. Only one study described the blind method. Allocation concealment and selective reporting results were not mentioned in the 11 studies.

### Clinical Outcomes

#### Total Effective Rate

**FIGURE 3:**
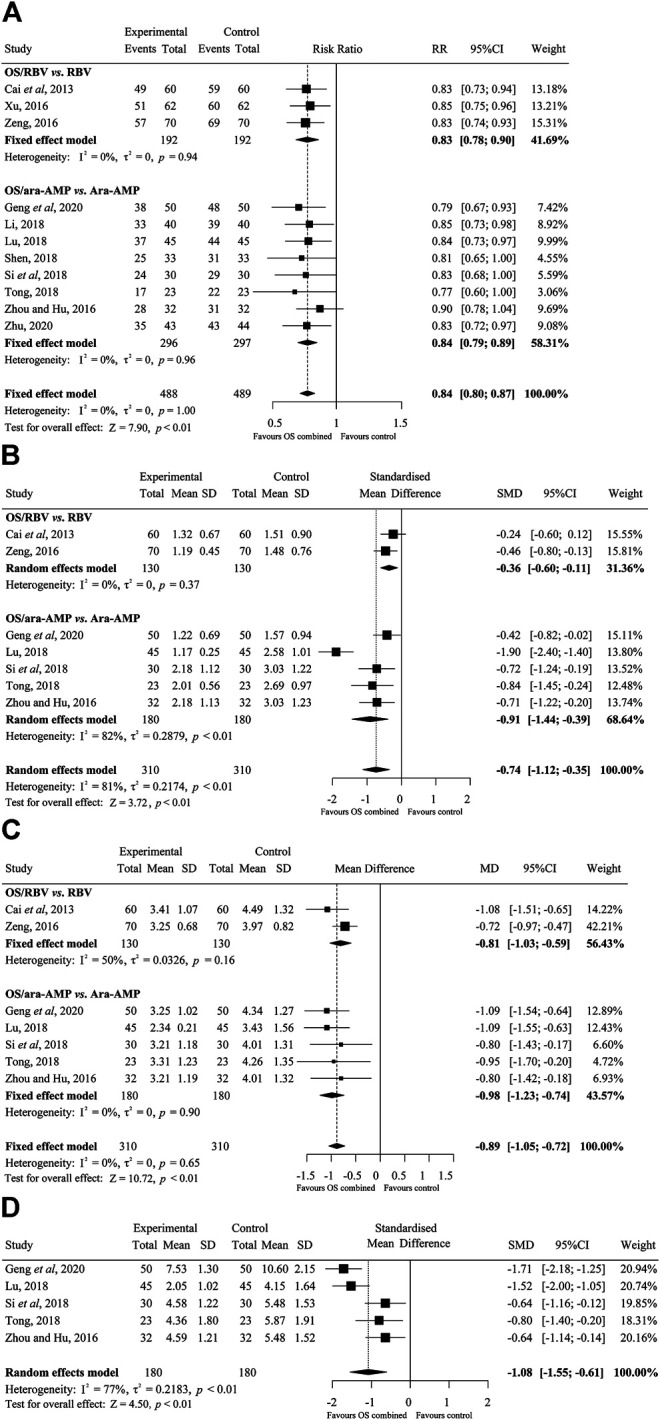
Forest plots of the efficacy of oseltamivir **(A)** Total effective rate, **(B)** fever clearance time, **(C)** rash regression time, and **(D)** clinical cure time. M-H, Mantel-Haenszel; CI, confidence interval; df, degrees of freedom; SD, standard difference; IV, inverse variance.

Sufficient data on total effective rate were reported in the 11 trials. The fixed effects model was used. Overall, the total effective rate in OS combined treatment group was significantly increased than that of the control group (RR, 0.84; 95% CI, 0.80–0.87; *I* ^*2*^, 0), and the difference was significant (*p* < 0.01). (Figure 3A).

#### Fever Clearance Time

There were seven studies on fever clearance time. The random effects model was used for the data analysis. The overall time to fever clearance in the OS combined treated group was significantly shorter than that of the control group (SMD, −0.74; 95% CI, −1.12 to −0.35; *p* < 0.01; *I* ^*2*^, 81%) (days). (Figure 3B).

#### Rash Regression Time

The results of the rash regression time are shown in Figure 3C. A total of seven studies were on the rash regression time. The fixed effects model was applied. The difference in rash regression time between treatment groups was statistically significant and OS treated patients recovered faster than that in the control groups (MD, −0.89; 95% CI, −1.05 to −0.72; *p* < 0.01; *I* ^*2*^, 0) (days).

#### Clinical Cure Time

Five studies that compared the cure time between OS/ara-AMP and ara-AMP groups presented data suitable for meta-analysis. The random effects model was applied for the analysis. There was statistically significant difference in clinical cure time between OS/ara-AMP and ara-AMP groups (SMD, −1.08; 95% CI, −1.55 to −0.61; *p* < 0.01; *I* ^*2*^, 77%) (days) (Figure 3D).

#### Adverse Reaction Rate

Four studies reported the incidence of adverse effects. Only one study showed that the incidence in the OS treated group was significantly lower than that of the control group. Other three studies reported no serious adverse reactions for the treatment period.

### Sensitivity Analysis and Publication Bias

**FIGURE 4:**
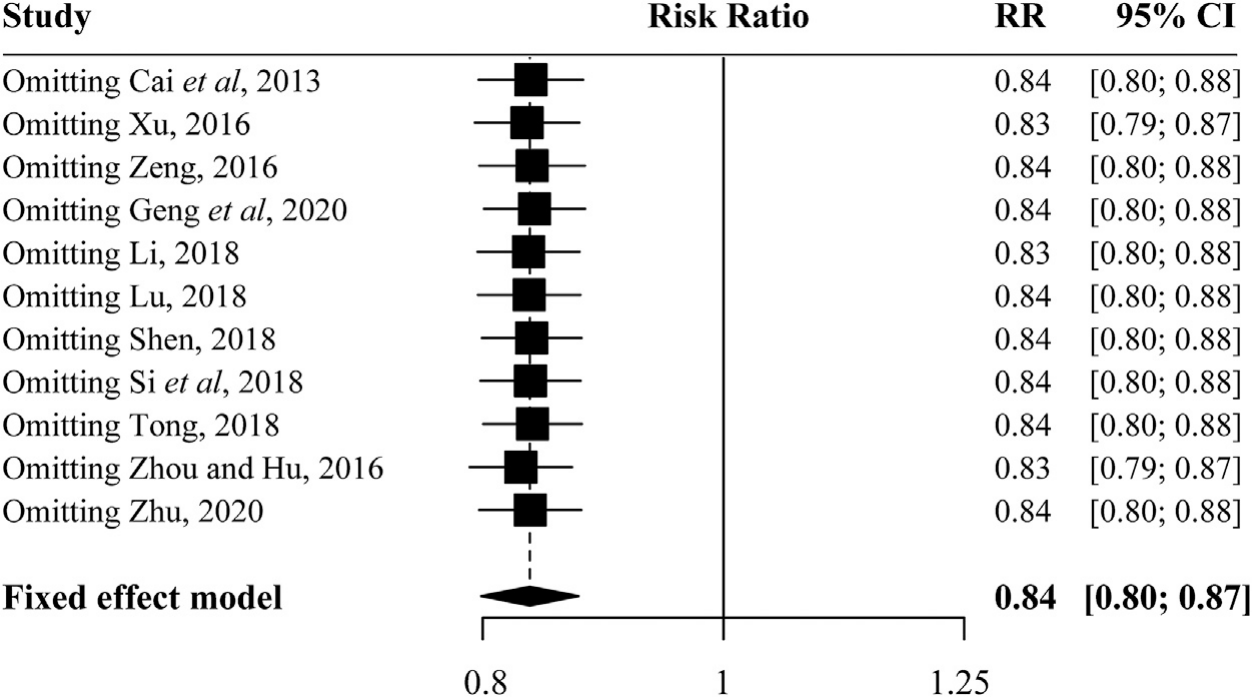
Sensitivity analysis for included studies.

**FIGURE 5:**
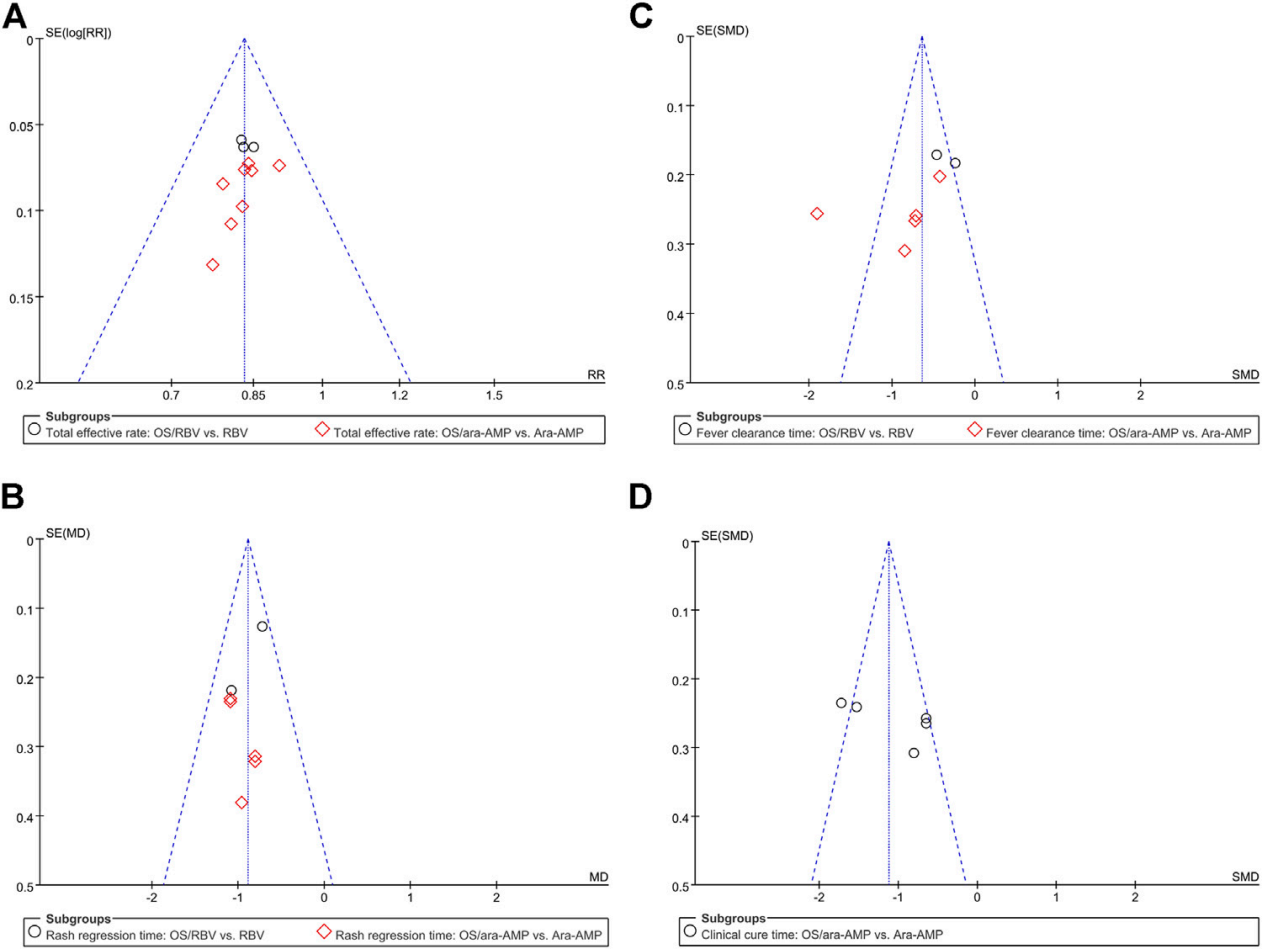
Bias analyses of publications **(A)** total effective rate, **(B)** fever clearance time, **(C)** rash regression time, and **(D)** clinical cure time.

The sensitivity analysis result showed that no eligible study influenced the pooled results materially (Figure 4). Moreover, the results of Begg’s tests showed that there was no evidence of publication bias in the present study (Figure 5).

## Discussion

HFMD is a common childhood illness caused by enteroviruses. It constitutes a substantial disease burden in East and Southeast Asia, and it is perennial in all parts of China (Dobson et al., 2015). Fever and skin lesions for one or two weeks may be distressing for both children and their parents. As of now, there is no specific anti-enterovirus drug available for the treatment of HFMD. If oseltamivir can shorten the duration and intensity of the illness, it may be used as an adjuvant therapy for HFMD. Our meta-analysis confirmed that oseltamivir combined therapy exhibited beneficial effect on HFMD, with higher effective rate, shorter fever clearance time, shorter rash regression time and shorter clinical cure time with no severe adverse reactions.

Oseltamivir is an oral prodrug which undergoes hydrolysis by hepatic esterases to form active oseltamivir carboxylate which acts by selective inhibition of influenza A and B viral neuraminidase. A lipophilic side chain of the active drug binds to the virus’ enzyme, blocking its ability to cleave sialic acid residues on the surface of the infected cell resulting in an inability to release progeny virions (Moscona, 2005). Sialic acid also known as neuraminic acid is usually linked to galactose or other sugar residues as an antenna of blood group antigens, tumor antigens, or viral receptors (Varki and Varki, 2007). EV71 is a major causative agent of HFMD that could result in high mortality of children when the central nervous system is infected by same (Solomon et al., 2010). Sialic-acid-linked glycan, which is abundantly expressed in the respiratory and gastrointestinal tracts, and dendritic cell-specific intercellular adhesion-molecule-3-grabbing non-integrin (CD209), has been identified as a receptor for EV71 (Lin et al., 2009; Yang et al., 2009). Cell surface sialic acids assist in the attachment of EV71 to host cells. Cell surface sialylation may be a key regulator of EV71 (Su et al., 2012). This sialic acid link could be a common pathway by which oseltamivir exerts it therapeutic effect in HFMD. In addition, oseltamivir also possesses the ability to cross the blood-brain barrier Adhisivam and Venkatesh (2015) and appears to be effective in the treatment of acute encephalitis caused by HFMD.

The present study has some limitations. First, the included studies were all from China, and they were published in Chinese and were not indexed in PubMed. HFMD is highly prevalent in Asian countries, especially in China. Clinical trials of HFMD could not be retrieved in PubMed, EMBASE, and Cochrane database. Second, the included studies did not specify the type of enterovirus and the severity of the enrolled patients. Thirdly, the studies examined were based on relatively small numbers of participants ranging from 23 to 70. Fourthly, the methodological quality of the clinical studies was generally poor. Only one study described the blind method. Allocation concealment and selective reporting results were not mentioned in the 11 studies. Finally, limited evidence is available on the potential molecular mechanism of action for oseltamivir in the treatment of HFMD. Further multi-centers from different country, large-scale, well-designed and functional studies are warranted to better explore the efficacy of oseltamivir against HFMD.

## Conclusion

This meta-analysis provides valuable evidence regarding the efficacy of oseltamivir for the treatment of HFMD. The results from the present study show that oseltamivir possesses high efficacy in adjuvant treatment of HFMD, without increasing the risk of adverse reactions. This study therefore provides a treatment option for HFMD in clinical practice.

## Data Availability

The original contributions presented in the study are included in the article, further inquiries can be directed to the corresponding authors.

## Abbreviations

ara-AMP, vidarabine monophosphate; CV-A16, coxsackievirus A16; EV71, enterovirus A71; HFMD, hand-foot-and-mouth disease; MD, mean differences; OS, oseltamivir; RBV, ribavirin; RCT, randomized clinical trial; RR, risk ratio; SMD, standardized mean difference.
